# Dystonia‐ataxia syndrome with permanent torsional nystagmus caused by ECHS1 deficiency

**DOI:** 10.1002/acn3.51025

**Published:** 2020-04-24

**Authors:** Dario Ronchi, Edoardo Monfrini, Sara Bonato, Veronica Mancinelli, Claudia Cinnante, Sabrina Salani, Andreina Bordoni, Patrizia Ciscato, Francesco Fortunato, Marianna Villa, Alessio Di Fonzo, Stefania Corti, Nereo Bresolin, Giacomo P. Comi

**Affiliations:** ^1^ Fondazione IRCCS Ca' Granda Ospedale Maggiore Policlinico Neurology Unit Milan Italy; ^2^ Dino Ferrari Center Department of Pathophysiology and Transplantation University of Milan Milan Italy; ^3^ Fondazione IRCCS Ca' Granda Ospedale Maggiore Policlinico Neuroradiology Unit Milan Italy; ^4^ Fondazione IRCCS Ca' Granda Ospedale Maggiore Policlinico Neuromuscular and Rare Diseases Unit Milan Italy

## Abstract

Biallelic mutations in *ECHS1*, encoding the mitochondrial enoyl‐CoA hydratase, have been associated with mitochondrial encephalopathies with basal ganglia involvement. Here, we describe a novel clinical presentation consisting of dystonia‐ataxia syndrome with hearing loss and a peculiar torsional nystagmus observed in two adult siblings. The presence of a 0.9‐ppm peak at MR spectroscopy analysis suggested the accumulation of branched‐chain amino acids. Exome sequencing in index probands identified two *ECHS1* mutations, one of which was novel (p.V82L). ECHS1 protein levels and residual activities were reduced in patients’ fibroblasts. This paper expands the phenotypic spectrum observed in patients with impaired valine catabolism.

## INTRODUCTION

*ECHS1* encodes the mitochondrial enoyl‐CoA hydratase which catalyzes the fourth degradation step of the branched‐chain amino acid (BCAA) valine but also takes part in the beta‐oxidation of short‐chain fatty acids, contributing to energy metabolism.^1^

Biallelic mutations in *ECHS1* (OMIM 612677) have been mainly associated with early‐onset Leigh‐like syndrome (LLS) presenting severe progressive encephalopathy and signs of mitochondrial dysfunction including epilepsy, optic atrophy and hearing loss.^2^ Cardiac abnormalities were also observed.^3^ Prognosis is poor, with the mean age at death of 69 months although patients reaching adulthood have been described.^4^ Additional clinical symptoms might include: *cutis laxa*,^5^ paroxysmal exercise‐induced dyskinesia^6^ and dystonia.^7^

Increased serum lactate levels and elevated urinary concentrations of 2‐methyl‐2,3‐dihydroxybutyrate and N‐acetyl‐S‐(2‐carboxypropyl)cysteine have been reported.^8^ Reduced activities of pyruvate dehydrogenase and respiratory chain complexes have been linked with the accumulation of acryloyl‐CoA and methacrylyl‐CoA, originating from incomplete valine catabolism.^9^ However, these abnormalities are detectable only in a subset of severe clinical courses.

## PATIENTS AND METHODS

The study was approved by the institutional review board of the Fondazione IRCCS Ca’ Granda Ospedale Maggiore Policlinico. The patients provided written informed consent for all aspects of the study.

**Figure 1:**
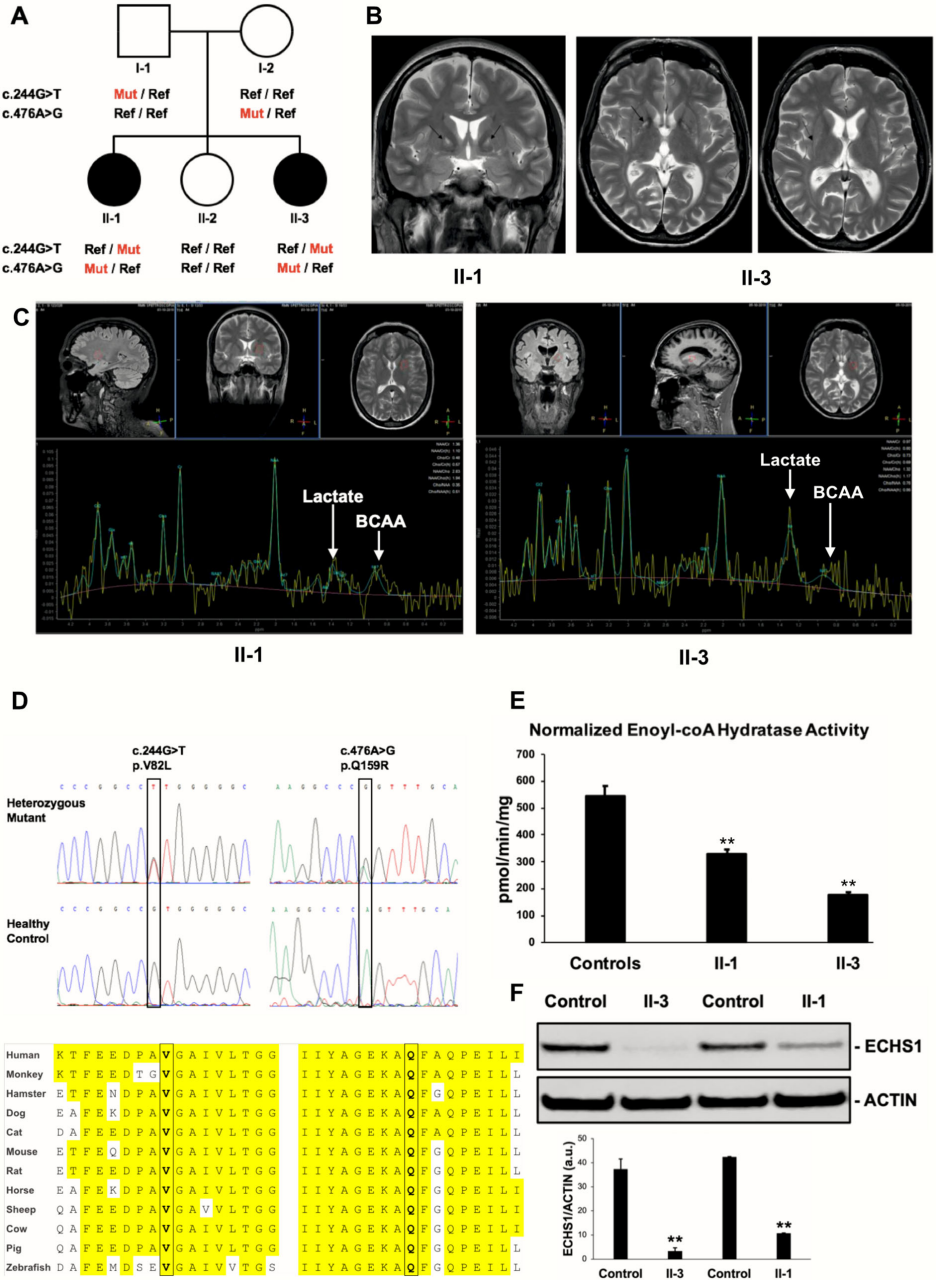
Clinical, genetic and biochemical findings. (A) Pedigree and genotypes of the family investigated. (B) MRI scans showing symmetric T2‐hyperintense alterations localized (black arrows) in the *globi pallidi* (II‐1 and II‐3) and the posterior part of the putamina (II‐3). (C) MR spectroscopy analysis of the lenticular lesions in Subjects II‐1 and II‐3 showing (white arrows) an increased peak at 1.3 ppm (Lactate) and the presence of a 0.9 ppm peak (BCAA). (D) Electropherogram showing the *ECHS*1 nucleotide substitutions detected in our patients and resulting in conserved amino acid changes. (E) Biochemical analysis of Enoyl‐coA hydratase activities in patients’ and control (*n* = 4) fibroblasts. Values are expressed as pmol/min/mg after normalization to citrate synthase activity and presented as mean ± standard deviation (three replicates, **P < 0.05). (F) Western Blot analysis of ECHS1 protein signals normalized to ACTIN in patients and control fibroblasts. Values are expressed as arbitrary units (a.u.) and presented as mean ± standard deviation of three independent experiments (**P < 0.05).

The parents (Subjects I‐1 and I‐2) of the two affected siblings (II‐1 and II‐3) were neurologically healthy and not related (Fig. 1A). A third sibling (II‐2) did not display any sign of neurological involvement. Both probands were born late preterm after an uneventful pregnancy.

Subject II‐1 presented early motor delay with difficulty in fine motor skills, unstable gait, and frequent falls. Mild speech delay was present and was attributed to congenital sensorineural hearing loss. From the age of 4 years, she developed a permanent nystagmus. Motor deficits presented a slowly progressive course. The patient had normal intelligence and she graduated from college. She presented at our hospital at the age of 32 years, showing a prominent unceasing torsional nystagmus (Video S1) associated with hypoacusia, cerebellar dysarthria, dysmetria, and widespread moderate to severe increase in the muscular tone of the four limbs, and bilateral Babinski sign. An unsteady dystonic‐ataxic gait was observed.

Subject II‐3 displayed a normal psychomotor development. Hypoacusia was present since birth. At the age of 5 years, a permanent torsional nystagmus appeared. Intelligence was normal and she graduated from college. She presented at our hospital at the age of 24 years, presenting with unceasing torsional nystagmus. Associated clinical features were mild cerebellar dysarthria, mild dysmetria, intermittent dystonic movement of the left foot, and bilateral Babinski sign. Mobility was normal, although the tandem gait test was performed with difficulty.

In both probands, brain MRI showed symmetric T2‐hyperintense alterations localized in the *globi pallidi* and the posterior part of the *putamina*, reminiscent of Leigh‐like lesions. A mild atrophy of cerebellar vermis was also present (Fig. 1B). MR spectroscopy analysis of the lenticular lesions revealed a reduction of the N‐acetylaspartate (NAA) peak, an increased lactic acid peak, and the presence of a 0.9‐ppm peak, corresponding to elevated BCAAs Valine, Leucine and Isoleucine (Fig. 1C).

Electroencephalography (EEG) showed bilateral recurrent bursts of sharp waves in Subject II‐3. EEG background was well‐formed. Electromyography (EMG) and nerve conduction studies (NCS) displayed chronic myogenic changes in Subject II‐1. Motor evoked potentials (MEPs) demonstrated a moderate slowing of the central conduction velocity in both the upper and lower limbs in Subject II‐3. MEPs were suggestive of corticospinal tract dysfunction. Indeed, the increased muscular tone was attributed to a combination of dystonia and spasticity.

Muscle biopsies of the left biceps brachii, performed in both siblings, showed only few COX‐negative fibers in Subject II‐1. No cardiological abnormalities were found (electrocardiography and echocardiography). Serum lactic acid was mildly increased. Urine organic acid profiles were normal. Ocular fundus examination was normal.

Experimental methods are described in Supplementary Data.

## RESULTS

Exome scans in the affected sisters detected two missense mutations in *ECHS1* (NM_004092.4) (Figures S1 and S2). The previously described^3^ c.476A> G mutation (chr10:135,182,465T> C) results in the amino acid substitution p.Q159R. The c.244G> T variant (chr10:135,184,106C> A) is novel and produces a p.V82L change, predicted as deleterious (Fig. 1D and Figure S3). Segregation test confirmed the recessive inheritance (Figure S4).

ECHS1 enzyme activity was spectrophotometrically measured in cultured fibroblasts^10^ and found markedly reduced in patients (mean values 253.65 ± 107.00 pmol/min/mg) compared to controls (546.13 ± 36.30 pmol/min/mg, *P* < 0.01, Fig. 1E). Similarly, steady‐state levels of the mitochondrial ECHS1 protein (Figure S5) were found reduced in patients compared to controls (*P* < 0.01): residual protein levels were 26.5% and 8.25% in Subjects II‐1 and II‐3, respectively (Figure 1F and Figure S6).

**Figure 2:**
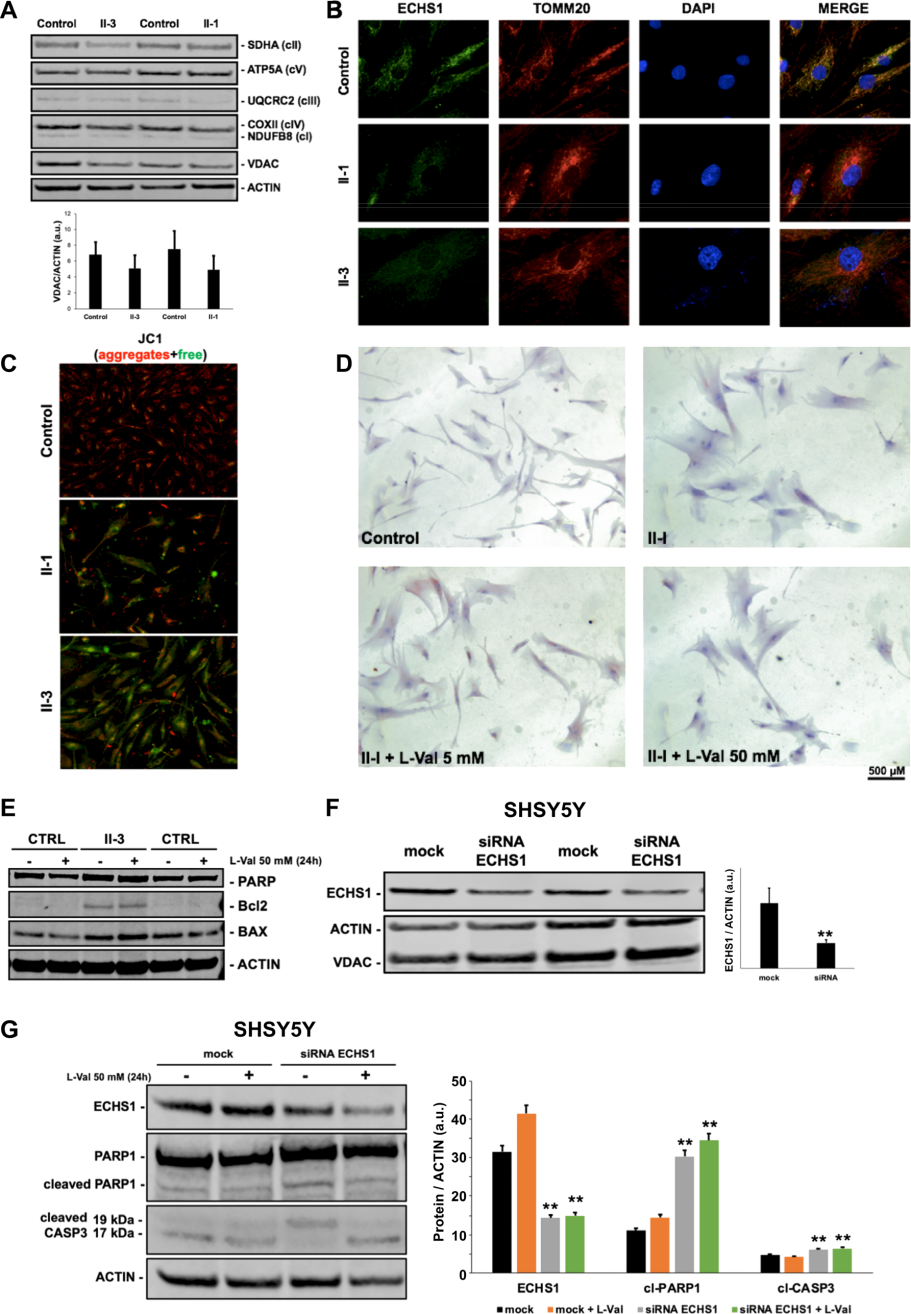
Immunocytochemical and protein studies in cellular models of ECHS1 deficiency. (A) Western Blot analysis of representative subunits of mitochondrial respiratory chain. Histograms show densitometric analysis (arbitrary units, a.u.) expressed as mean ± standard deviation of three experiments. No difference was observed between patients’ and controls fibroblasts. (B) Immunocytochemical studies addressing mitochondrial content and localization (TOMM20, green) supported the mitochondrial localization of ECHS1 and did not show any clear difference between patient’s and controls fibroblasts. (C) JC1 staining of free cytoplasmic aggregates (green) in patients’ fibroblasts indicative of mild dissipation of mitochondrial membrane potential. (D) Oil Red O (ORO) staining to detect intracellular lipids (cytosolic red dots) did not show any difference between patients and controls cells in basal conditions or after supplementation of L‐Valine in culture medium (5 and 50 mmol/L for 24 h). (E) Representative Western blot analysis of apoptotic markers PARP1, Bcl2 and BAX in fibroblasts of Subjects II‐3 and healthy controls in basal condition or after L‐Valine supplementation (50 mmol/L, 24 h). Actin was used as a loading control. (F) Downregulation of ECHS1 expression in SHSY5Y after siRNA‐based interference for 48 hours compared to mock‐transfected condition (two replicates, **P < 0.05). (G) Representative Western blot analysis of total and cleaved (cl‐) apoptotic markers PARP1 and CASP3 in mock‐transfected or silenced (siRNA ECHS1) SHSY5Y cells. Histograms show densitometric analysis (arbitrary units) expressed as mean ± standard deviation of three experiments (**P < 0.05) performed in basal conditions or after L‐Valine supplementation (50 mmol/L, 24 h). ACTIN was used as a loading control.

No significant difference in the expression of representative mitochondrial respiratory chain subunits or the marker of mitochondrial content PORIN was observed (Fig. 2A and Figure S7). Immunocytochemical studies in fibroblasts demonstrated the mitochondrial localization of ECHS1 immunoreactivity, as determined by colocalization with TOMM20, and confirmed the reduction of ECHS1 expression in the two patients. TOMM20 staining did not display alterations in the distribution of mitochondria (Fig. 2B). The evaluation of mitochondrial membrane potential showed slight changes in ECHS1‐mutated cells (Fig. 2C and Figure S8). Red‐oil staining of patients’ cells^11^ did not display lipid accumulation (Fig. 2D and Figure S9), reflecting the negative results of muscle biopsy (not shown).

The expression of apoptotic markers BAX and cleaved PARP1 was unchanged in mutated and control fibroblasts, even after L‐valine supplementation (Fig. 2E). The experiment was repeated in human neuroblastoma SHSY5Y cells after ECHS1‐downregulation obtained by transient RNA interference and confirmed at protein level (30% compared to mock‐transfected cells) (Fig. 2F). In ECHS1‐silenced neuronal cells, activation of apoptosis was suggested by the detection of cleaved PARP1 and Caspase 3 forms before and after L‐valine supplementation (Fig. 2G),

## DISCUSSION

We described two siblings with ECHS1‐deficiency associated with a novel dystonia‐ataxia syndrome allowing survival into adulthood. Besides bilateral hearing loss, our patients also showed an impressive rotatory nystagmus and, in the older sister, a severe muscle hypotrophy. Cardiac and optic nerve involvement were ruled out.

To our knowledge, our patients are the most long‐living ECHS1‐mutated subjects reported so far. Heterogenous clinical presentations are not uncommon in primary mitochondrial diseases, even in the presence of the same genotype. The p.Q159R has been previously described in either the homozygous or compound heterozygous state in clinically affected patients mainly showing LLS with early‐infancy onset and premature death.^12^ Haack and co‐authors described a 31‐year old woman with spastic tetraparesis who had lost deambulation at the age of 9.5. Additional clinical symptoms included optic atrophy, developmental delay, epilepsy and hearing loss. The second allele showed a missense mutation at codon 77 which allowed a partial conservation of ECHS1 activity.^3^ In our probands, the second mutation is located nearby (codon 82) and residual protein and activity levels are overall higher compared to severe pediatric presentations. The review of published cases for which residual ECHS1 activity levels in fibroblasts were available^2^, ^3^, ^9^, ^13^, ^14^, ^15^ suggests a positive correlation between enzyme activity and survival (Figure S10, Pearson’s correlation coefficient *r* = 0.88, *P* < 0.05). These observations are meaningful for prognosis and the identification of biochemical thresholds to monitor experimental therapies aiming to rescue ECHS1 activity.

Increased levels of serum pyruvate, lactate and Krebs cycle metabolites point to a deficit in energy metabolism, but these unspecific findings are not consistently observed in all *ECHS1*‐mutated patients, including ours. We failed to observe clear evidences of mitochondrial respiratory chain dysfunction which is limited to the presence of few COX‐negative fibers in the muscle of the elder sister. Similarly, no accumulation of lipids was found at the tissue and cellular levels. Therefore, ECHS1‐mutated fibroblasts did not show prominent alterations of mitochondrial respiratory chain or fatty acids catabolism, exculpating these pathways as the main pathogenetic drivers of ECHS1 deficiency.

A positive MRI scan with basal ganglia involvement has been documented in all the presentations due to *ECHS1* mutations, despite clinical symptoms and course, not differently from other disorders featuring Leigh or Leigh‐like syndromes. Brain spectroscopy was more specific and disclosed in both of our patients a peak corresponding to BCAA. Therefore, brain spectroscopy could be used to accelerate diagnosis and to validate genetic findings in additional patients for which biochemical investigations are negative or unavailable.

The elucidation of the pathogenetic cascade originating from ECHS1‐deficiency is important for the design of therapies. For example, ROS scavenging might be ineffective if respiratory chain dysfunction is a secondary event, while dietary restrictions to limit BCAA intake could have minimal effects on BCAA accumulation in the central nervous system.^16^ In this regard, we observed the activation of programed cellular death only when ECHS1 deficiency was experimentally achieved in a neuronal model. Further studies in patient‐specific neuronal cells are mandatory to explore the mechanisms underlining the cell‐type selectivity of ECHS1 deficiency.

The overlapping clinical features observed in our patients and in two subjects harboring mutations in HIBCH, the enzyme acting downstream ECHS1 in valine catabolism, suggest a key role for BCAA metabolism in brain biochemistry.^17^ Indeed, ECHS1 has been recently proposed as an intracellular sensor of nutrients able to influence mammalian target of Rapamycin (mTOR) signaling and apoptosis.^18^ These findings refocus ECHS1‐deficiency, and the consequent increase of BCAA levels, in the context of global intracellular metabolism. Pharmacological manipulation of mTOR might alleviate the metabolic effects of ECHS1‐deficiency paralleling the results obtained in other genetic forms of mitochondrial‐related neurodegeneration,^19^ including Leigh Syndrome.^20^

## Conflict of Interest

No conflicts of interest to disclose.

## Author Contributors

SB and MV performed a clinical assessment of the probands. DR and EM performed genetic and molecular studies. VM and SS established cell cultures and performed immunocytochemical analysis. AB and FF performed protein and enzymatic studies. PC performed lipid analysis. CC supervised neuroradiological data collection and analysis. ADF, SC, NB, GPC: manuscript revision. DR and EM were responsible for drafting the manuscript and preparing figures.

## Supporting information


**Figure S1.** Prioritization workflow of NGS data
**Figure S2.** IGV screenshots of ECHS1 mutations
**Figure S3.** Prediction of pathogenicity and allele frequency of ECHS1 mutations
**Figure S4.** Segregation studies and cDNA analysis in the investigated family.
**Figure S5.** Protein studies addressing ECHS1 subcellular localization.
**Figure S6.** Western blot analysis of ECHS1 in patients/control fibroblasts.
**Figure S7.** Western blot analysis of representative mitochondrial respiratory chain subunits in patients/control fibroblasts.
**Figure S8.** JC1‐staining in patients’ and controls fibroblasts.
**Figure S9.** ORO staining in control and patients’ fibroblasts.
**Figure S10.** Correlation between residual ECHS1 activities and age at onset in ECHS1‐mutated subjects so far described.

**Video S1.** Video displaying unceasing torsional nystagmus in Subject II‐1.

## References

[1] Sharpe A , McKenzie M . Mitochondrial fatty acid oxidation disorders associated with short‐chain Enoyl‐CoA Hydratase (ECHS1) deficiency. Cells 2018;7:46. 10.3390/cells7060046 PMC6025059 29882869

[2] Peters H , Buck N , Wanders R , et al. ECHS1 mutations in Leigh disease: A new inborn error of metabolism affecting valine metabolism. Brain 2014;137:2903–2908. 25125611 10.1093/brain/awu216

[3] Haack TB , Jackson CB , Murayama K , et al. Deficiency of ECHS1 causes mitochondrial encephalopathy with cardiac involvement. Ann Clin Transl Neurol 2015;2:492–509. 26000322 10.1002/acn3.189 PMC4435704

[4] Aretini P , Mazzanti CM , La Ferla M , et al. Next generation sequencing technologies for a successful diagnosis in a cold case of Leigh syndrome. BMC Neurol 2018;18:1–6. 30029642 10.1186/s12883-018-1103-7 PMC6054728

[5] Balasubramaniam S , Riley LG , Bratkovic D , et al. Unique presentation of cutis laxa with leigh‐like syndrome due to ECHS1 deficiency. J Inherit Metab Dis 2017;40:745–747. 28409271 10.1007/s10545-017-0036-4

[6] Mahajan A , Constantinou J , Sidiropoulos C . ECHS1 deficiency‐associated paroxysmal exercise‐induced dyskinesias: case presentation and initial benefit of intervention. J Neurol 2017;264:185–187. 28039521 10.1007/s00415-016-8381-z

[7] Olgiati S , Skorvanek M , Quadri M , et al. Paroxysmal exercise‐induced dystonia within the phenotypic spectrum of ECHS1 deficiency. Mov Disord 2016;31:1041–1048. 27090768 10.1002/mds.26610

[8] Peters H , Ferdinandusse S , Ruiter JP , et al. Metabolite studies in HIBCH and ECHS1 defects: implications for screening. Mol Genet Metab 2015;115:168–173. 26163321 10.1016/j.ymgme.2015.06.008

[9] Ferdinandusse S , Friederich MW , Burlina A , et al. Clinical and biochemical characterization of four patients with mutations in ECHS1. Orphanet J Rare Dis 2015;10:1–15. 26081110 10.1186/s13023-015-0290-1 PMC4474341

[10] Stern JR , Del Campillo A , Raw I . Enzymes of fatty acid metabolism. I. General introduction; crystalline crotonase. J Biol Chem 1956;218:971–983. 13295247

[11] Kinkel AD , Fernyhough ME , Helterline DL , et al. Oil red‐O stains non‐adipogenic cells: a precautionary note. Cytotechnology 2004;46:49–56. 19003258 10.1007/s10616-004-3903-4 PMC3449473

[12] Ganetzky RD , Bloom K , Ahrens‐Nicklas R , et al. ECHS1 deficiency as a cause of severe neonatal lactic acidosis. JIMD Rep 2016;30:33–37. 26920905 10.1007/8904_2016_538 PMC5110442

[13] Yamada K , Aiba K , Kitaura Y , et al. Clinical, biochemical and metabolic characterisation of a mild form of human short‐chain enoyl‐CoA hydratase deficiency: Significance of increased n‐acetyl‐s‐(2‐carboxypropyl)cysteine excretion. J Med Genet 2015;52:691–698. 26251176 10.1136/jmedgenet-2015-103231

[14] Fitzsimons PE , Alston CL , Bonnen PE , et al. Clinical, biochemical, and genetic features of four patients with short‐chain enoyl‐CoA hydratase (ECHS1) deficiency. Am J Med Genet Part A 2018;176:1115–1127. 29575569 10.1002/ajmg.a.38658 PMC5947294

[15] Bedoyan JK , Yang SP , Ferdinandusse S , et al. Lethal neonatal case and review of primary short‐chain enoyl‐CoA hydratase (SCEH) deficiency associated with secondary lymphocyte pyruvate dehydrogenase complex (PDC) deficiency. Mol Genet Metab 2017;120:342–349. 28202214 10.1016/j.ymgme.2017.02.002 PMC5382105

[16] Griffin JWD , Bradshaw PC . Amino acid catabolism in Alzheimer’s disease brain: Friend or Foe? Oxid Med Cell Longev 2017;2017:5472792. 28261376 10.1155/2017/5472792 PMC5316456

[17] Schottmann G , Sarpong A , Lorenz C , et al. A movement disorder with dystonia and ataxia caused by a mutation in the HIBCH gene. Mov Disord 2016;31:1733–1739. 27400804 10.1002/mds.26704

[18] Zhang YK , Qu YY , Lin Y , et al. Enoyl‐CoA hydratase‐1 regulates mTOR signaling and apoptosis by sensing nutrients. Nat Commun 2017;8:464. 28878358 10.1038/s41467-017-00489-5 PMC5587591

[19] Zheng X , Boyer L , Jin M , et al. Alleviation of neuronal energy deficiency by mTOR inhibition as a treatment for mitochondria‐related neurodegeneration. Elife 2016;5:1–23. 10.7554/eLife.13378 PMC4846388 27008180

[20] Johnson SC , Yanos ME , Kayser EB , et al. mTOR inhibition alleviates mitochondrial disease in a mouse model of Leigh syndrome. Science 2013;342:1524–1528. 24231806 10.1126/science.1244360 PMC4055856

