# Supplementary material for: Dystonia‐ataxia syndrome with permanent torsional nystagmus caused by ECHS1 deficiency
Source: Ann Clin Transl Neurol. 2020 Apr 24;7(5):839–45. doi: 10.1002/acn3.51025 (PMC7261751; doi:10.1002/acn3.51025)
Supplement: Supplementary file 1 — Figure S1. Prioritization workflow of NGS data Figure S2. IGV screenshots of ECHS1 mutations Figure S3. Prediction of pathogenicity and allele frequency of ECHS1 mutations Figure S4. Segregation studies and cDNA analysis in the investigated family. Figure S5. Protein studies addressing ECHS1 subcellular localization. Figure S6. Western blot analysis of ECHS1 in patients/control fibroblasts. Figure S7. Western blot analysis of representative mitochondrial respiratory chain subunits in patients/control fibroblasts. Figure S8. JC1‐staining in patients’ and controls fibroblasts. Figure S9. ORO staining in control and patients’ fibroblasts. Figure S10. Correlation between residual ECHS1 activities and age at onset in ECHS1‐mutated subjects so far described. [file ACN3-7-839-s001.pdf]

Dystonia-ataxia syndrome with permanent torsional nystagmus caused by ECHS1 deficiency

SUPPLEMENTARY DATA

**Supplementary Materials and Methods**	
**Supplementary Video**	Video displaying unceasing torsional nystagmus in Subject II-1.
**Supplementary Figure 1**	Prioritization workflow of NGS data.
**Supplementary Figure 2**	IGV screenshots of ECHS1 mutations
**Supplementary Figure 3**	Prediction of pathogenicity and allele frequency of ECHS1 mutations.
**Supplementary Figure 4**	Segregation studies and cDNA analysis in the investigated family.
**Supplementary Figure 5**	Protein studies addressing ECHS1 subcellular localization.
**Supplementary Figure 6**	Western blot analysis of ECHS1 in patients/control fibroblasts.
**Supplementary Figure 7**	Western blot analysis of representative mitochondrial respiratory chain subunits in patients/control fibroblasts.
**Supplementary Figure 8**	JC1-staining in patients' and controls fibroblasts.
**Supplementary Figure 9**	ORO staining in control and patients' fibroblasts.
**Supplementary Figure 10**	Correlation between residual ECHS1 activities and age at onset in ECHS1-mutated subjects so far described.
**References cited in the Supplementary Data section**	

SUPPLEMENTARY MATERIALS AND METHODS

Neuroimaging

We performed Magnetic Resonance Imaging (MRI) using a 3 tesla scanner (Philips Achieva), acquiring morphological T1 and T2 images with turbo field echo (TFE) and fluid attenuated inversion recovery (FLAIR) 3D sequences, turbo spin echo (TSE) 2D sequences, completed with spectroscopy studies (MRS) using a single voxel placement in basal ganglia with a point RESolved spectroscopy sequence (PRESS) with a TE of 144 e 35 ms each.

Genetic Studies

Exome libraries were enriched using the SureSelect CRE v2 Kit (Agilent) and sequenced on a NextSeq550 instrument. FASTQ files were aligned to the human GRCh37.p11 genome build. Variants were prioritized by using Enlis genome software (https://www.enlis.com) and manually inspected by using IGV (https://software.broadinstitute.org/software/igv/). ECHS1 mutations were confirmed at genomic and cDNA (obtained from skin fibroblasts RNA) level by Sanger sequencing. Supplementary Figure 1 provides additional description of NGS metrics and variants prioritization workflow.

Biochemical studies

The activity Enoyl-CoA of hydratase was measured spectrophotometrically in fibroblasts lysates as previously described [S1] following the decrease in absorbance at 263 nm for 5 minutes after addition of Crotonyl-S-CoA (82.5 μM). Residual activities were normalized to those of the mitochondrial matrix enzyme citrate synthase. ECHS1 protein levels were evaluated in fibroblasts subcellular fractions or total lysates in a 4-12% SDS-PAGE by using a mouse monoclonal antibody (Proteintech 66117, 1:5000). Licor Odissey was used for image acquisition and analysis. Actin (Sigma A2066) was used for normalization purpose. A cocktail of antibodies was used to assess mitochondrial respiratory chain subunits (abcam ab110411, 1:1300). The mitochondrial PORIN (VDAC) was assayed by using a specific antibody (abcam ab15895, 1:1500). Additional antibodies were used to target BAX (Cell Signaling #2772, 1:1000), Bcl2 (abcam ab32124, 1:1000), cleaved CASP3 (Cell Signaling #9661, 1:800), PARP1 (Cell Signaling #9532, 1:1200).

Cellular studies

Skin fibroblasts were cultured in DMEM supplemented with 15% FBS. Neuroblastoma adherent cell line SH-SY5Y were maintained in DMEM/F12 nutrient mixture supplemented with 10% FBS, 1% Penicillin-Streptomycin, 2.5 mM L-Glutamine and 1x NEM NEAA. Immunocytochemistry (ICC) analysis of ECHS1 was performed as previously described [S2]. JC1 staining (Sigma CS0390) was performed according manufacturer instruction. Oil-Red O (ORO) staining was performed as described [S3] with little modification (reduction of washing steps time and incubation with ORO reagent for 1 hour).

ECHS1-silenced SH-SY5Y cells were obtained by using Lipofectamine® RNAi-MAX Transfection Protocol (Thermo Fisher Scientific, Invitrogen #13778075) to transfect TriFECTa® RNAi Kit (DsiRNAs: ECHS1-2 and ECHS1-3, IDT). Different conditions (single/double siRNA transfection, time of treatment, concentration of siRNA) were evaluated to find the optimal conditions for silencing of targets, preserving the recommended ratio between Lipofectamine e siRNA. 24 hours of co-administration of 30 nM ECHS1-2 and ECHS1-3 sequences resulted in a 70% transcript reduction without a significant increase in cellular mortality.

L-Valine (Sigma-Aldrich, Lot #SLBS4325) was added to culture medium at 5 mM and 50 mM final concentrations for variable times.

Statistical Analysis

Results from fibroblasts of the two patients were compared to three to five age matched-control subjects, according the experiment. The number of replicates is indicated in the figure legends.

A two-tailed Student's t test was used for statistical analyses. Graphs in figures represent mean ± s.d. with a statistical significance of $**p < 0.05$.

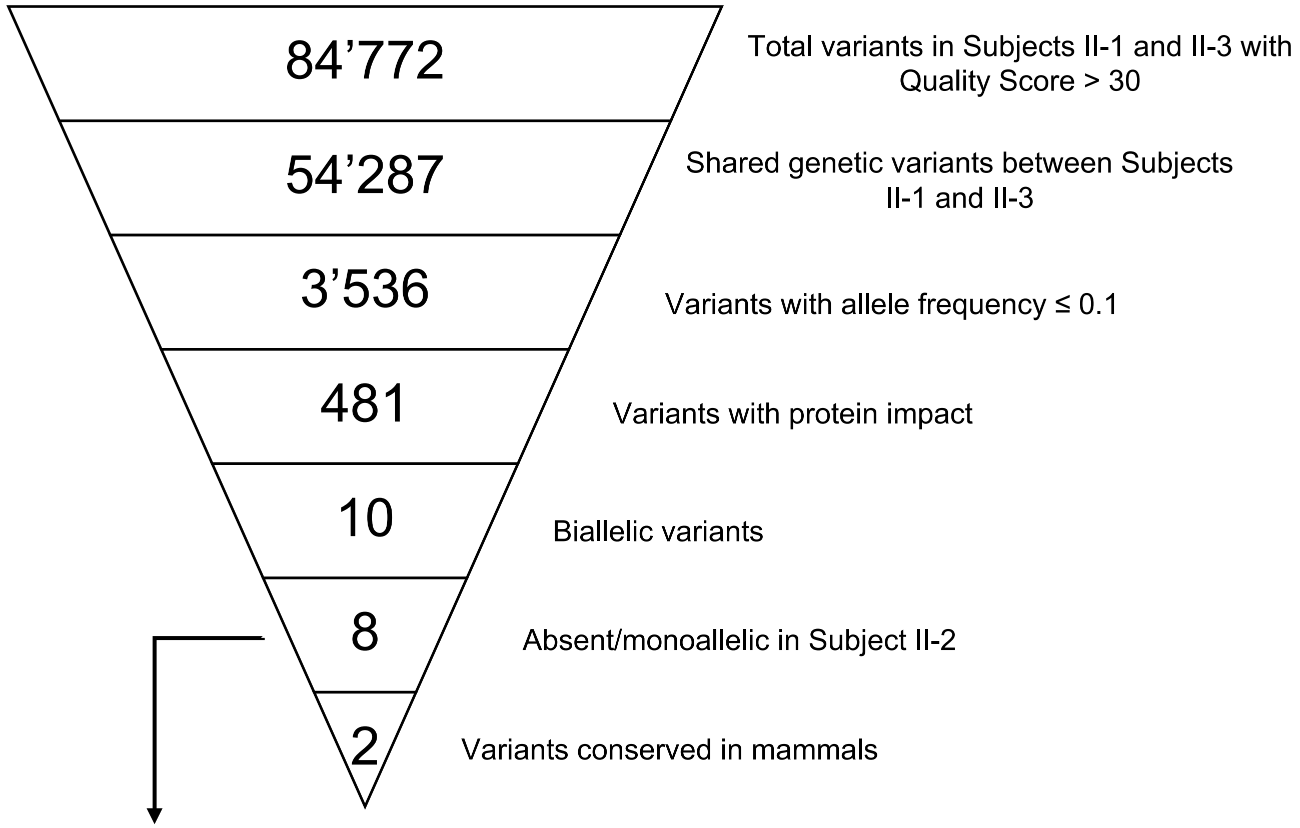

Chr.	Position	Ref.	Alt.	rs number	Gene	Variant type	Variant name	MAF	Mammalian Conservation
10	82012538	A	G	rs112413685	*AL359195.1*	MISSENSE	Q-19-R	0.04	No
10	82012556	T	C	rs532515417	*AL359195.1*	MISSENSE	F-25-S	0.10	No
**10**	**135184106**	**C**	**A**	**Novel**	***ECHS1***	**MISSENSE**	**V-82-L**	**0.00**	**Yes**
**10**	**135182465**	**T**	**C**	**rs375032130**	***ECHS1***	**MISSENSE**	**Q-159-R**	**0.01**	**Yes**
14	106110900	G	C	rs200481020	*IGHG2*	MISSENSE	F-76-L	0.10	No
14	106110904	T	C	rs201580297	*IGHG2*	MISSENSE	N-75-S	0.10	No
22	20708966	T	A	rs12172268	*FAM230A*	MISSENSE	V-233-D	0.00	No
22	20710700	T	C	rs62218181	*FAM230A*	MISSENSE	M-811-T	0.00	No

SUPPLEMENTARY FIGURE 1 Prioritization workflow of the variants identified by using Whole Exome Sequencing approach in this study. The table display the biallelic variants shared by affected patients.

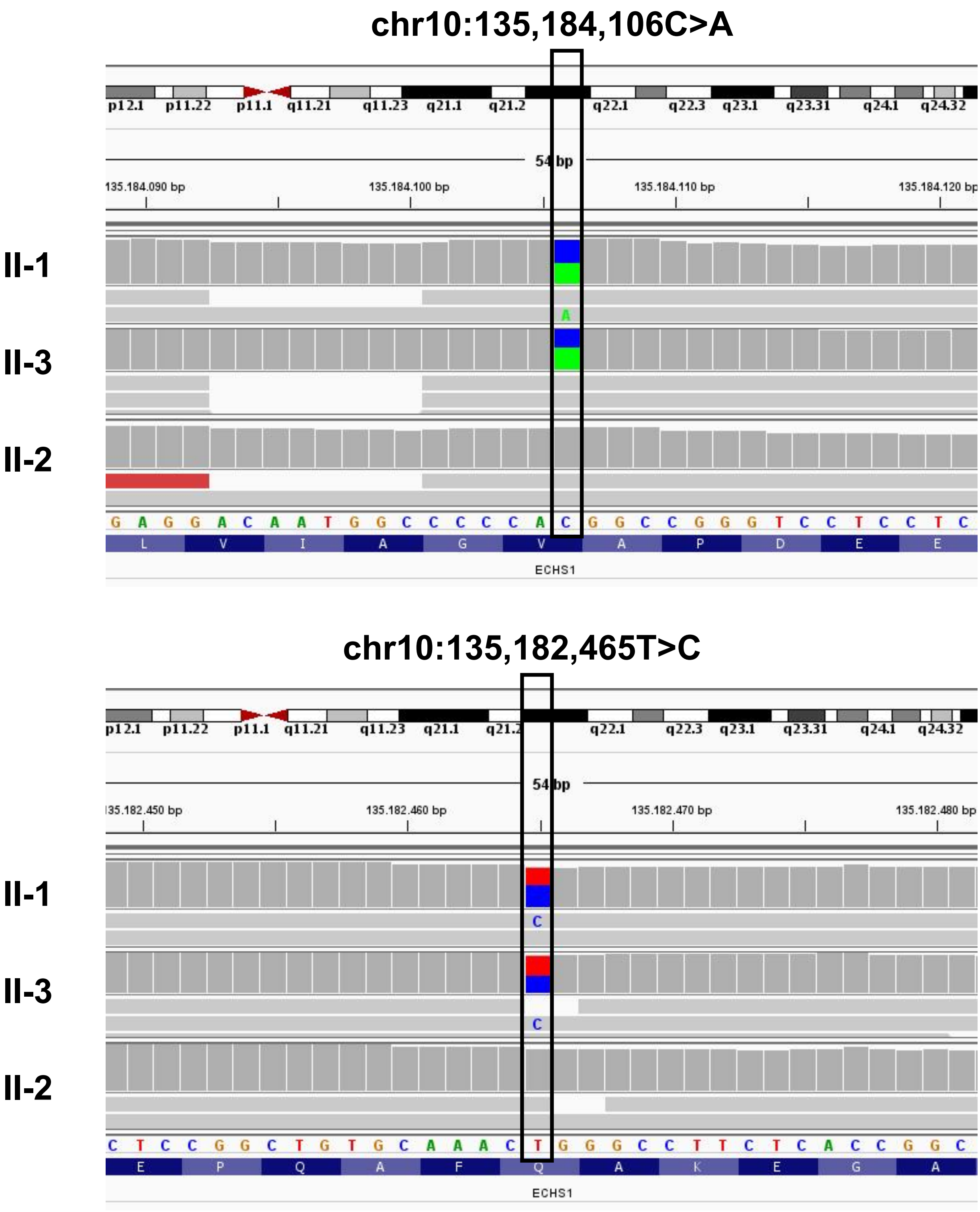

SUPPLEMENTARY FIGURE 2 Integrative Genomics Viewer screenshots showing the genomic variants called in *ECHS1* in the members of the family who undergo Whole Exome Sequencing.

	Mutation 1	Mutation 2
cDNA Position NM_004092.4	c.244G>T	c.476A>G
Protein Position NP_004083.3	p.Val82Leu (p.V82L)	p.Gln159Arg (p.Q159R)
Allele Frequency (EXAC)	0	0.0001162
Allele Frequency (gnomAD)	0	0.000106
Mutation Taster	PATHOGENIC	PATHOGENIC
CADD	21.2	32
Polyphen-2	DAMAGING	BENIGN
SIFT	NOT TOLERATED	TOLERATED

SUPPLEMENTARY FIGURE 3 Prediction of pathogenicity and allele frequency of the *ECHS1* missense mutations identified in this study.

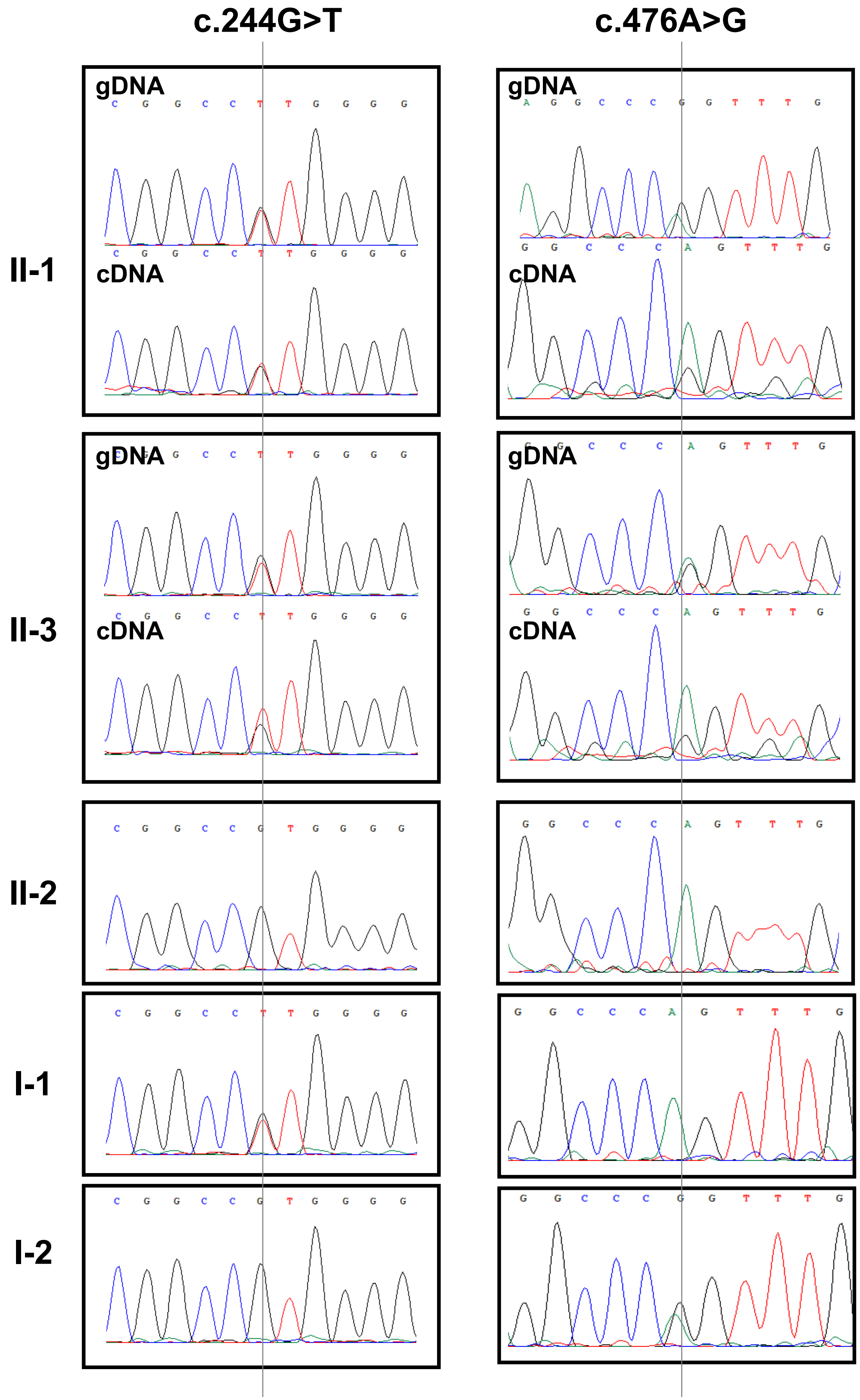

SUPPLEMENTARY FIGURE 4 Sequence electropherograms showing the segregation of ECHS1 mutations in available family members. For the probands, the variants were also checked at transcript level (RNA extracted from primary fibroblasts).

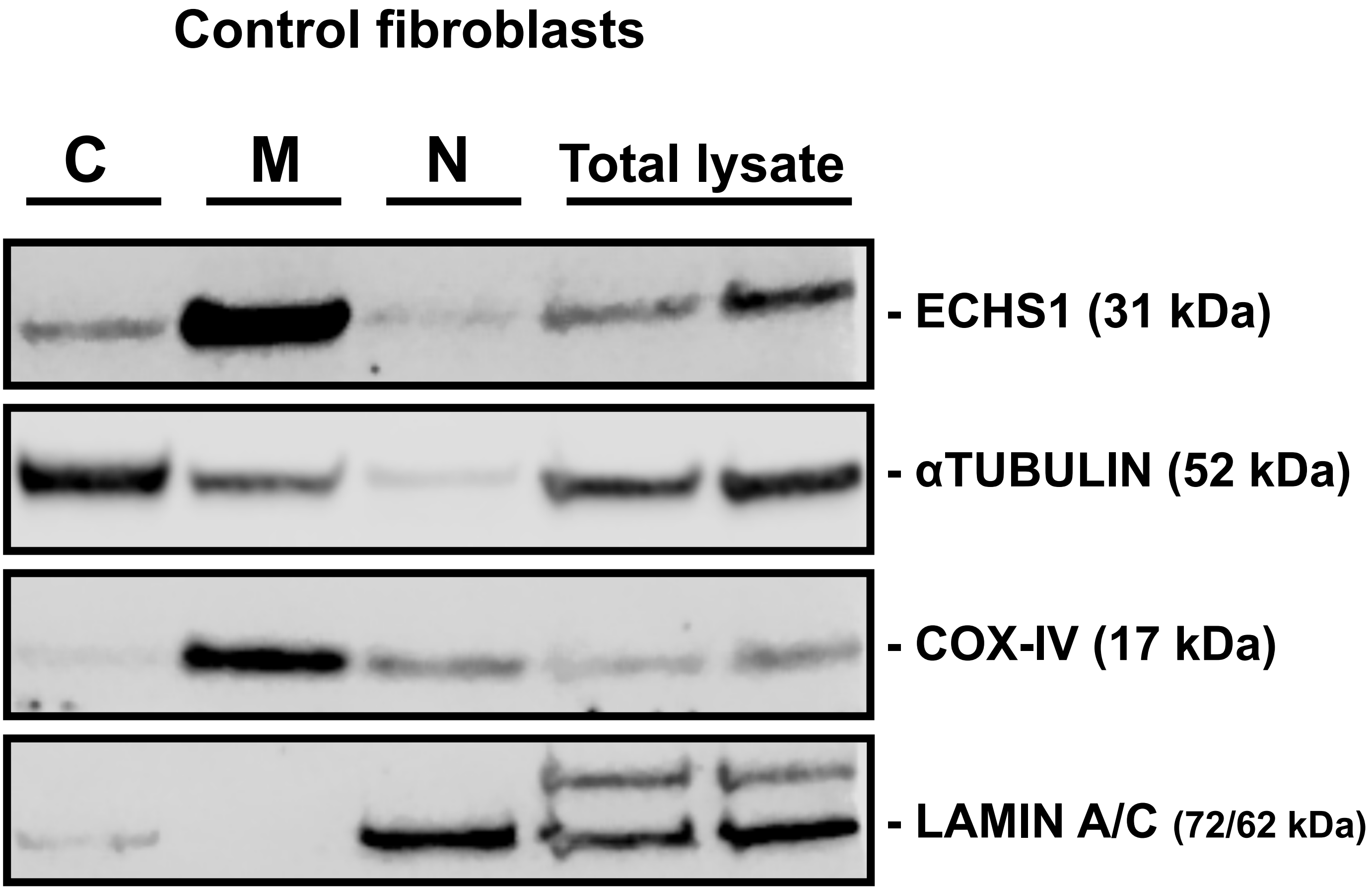

SUPPLEMENTARY FIGURE 5 Western blot analysis of ECHS1 localization in control fibroblasts. Appropriate markers were used to check the enrichment of the fractions obtained. (C: Cytosolic fraction, alpha-TUBULIN; M: Mitochondrial fraction, COX-IV; N: nuclear fraction, LAMIN A/C)

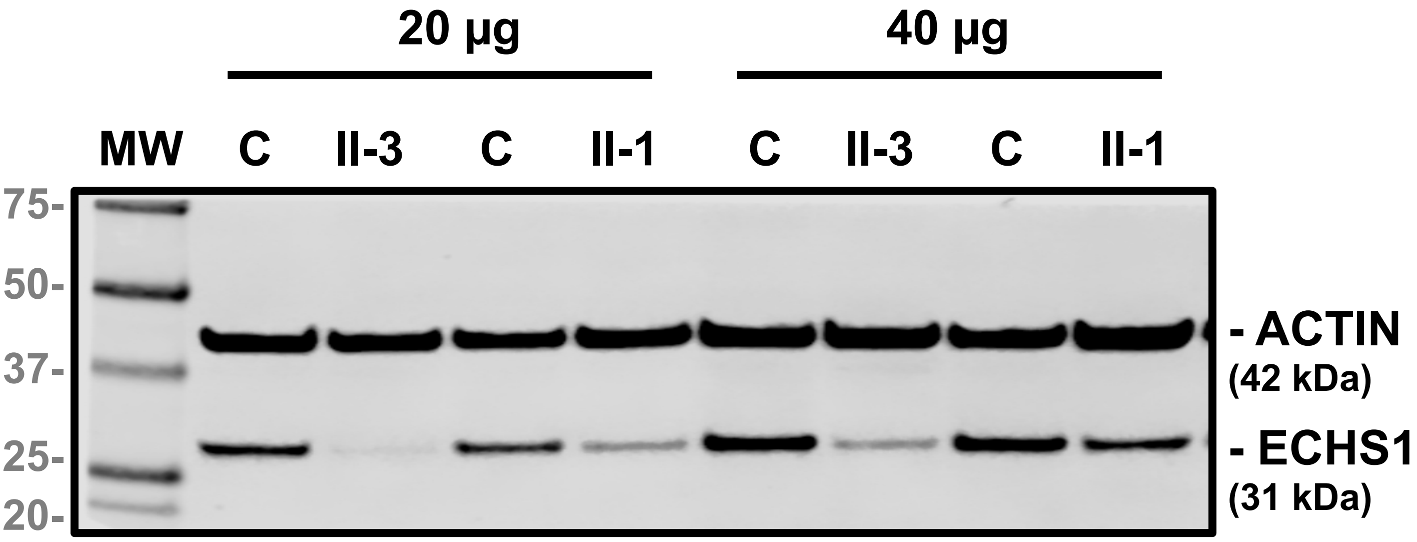

SUPPLEMENTARY FIGURE 6 Western blot analysis of ECHS1 in protein lysates of fibroblasts obtained from healthy controls (C) and ECHS1-Mutated Subjects (II-1, II-3). Two different amounts of proteins were loaded. ACTIN was used as loading control.

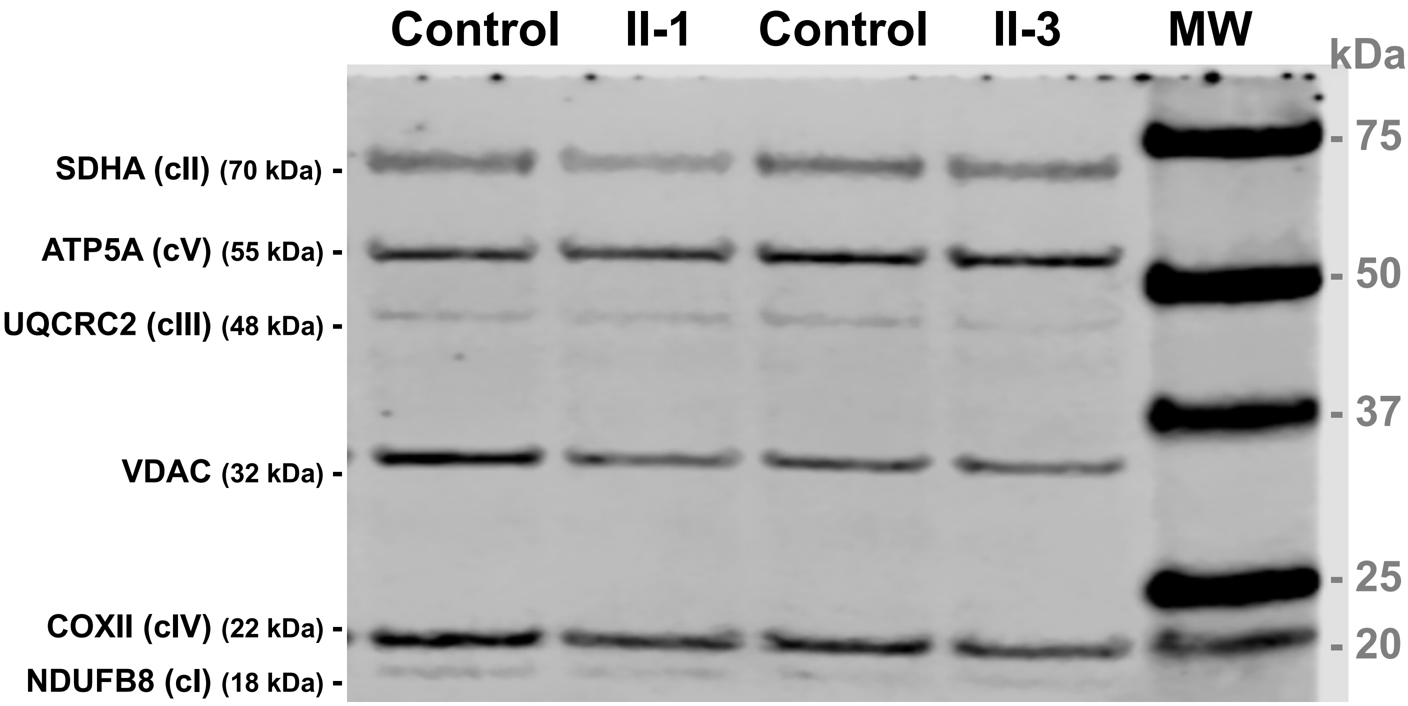

SUPPLEMENTARY FIGURE 7 Western blot analysis of representative Respiratory Chain Subunits of different complexes. The mitochondrial marker VDAC (PORIN) was used as loading control.

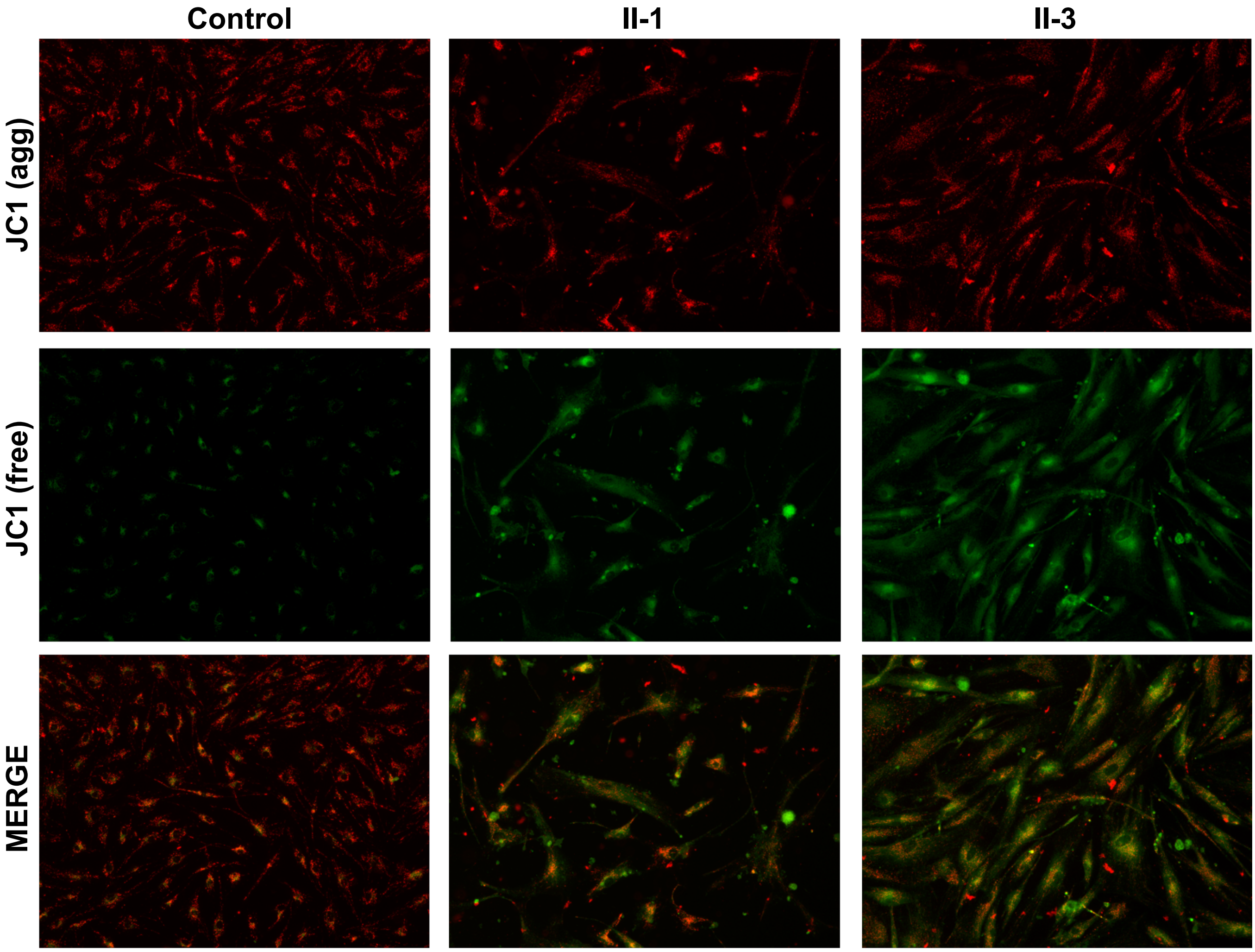

SUPPLEMENTARY FIGURE 8 JC1-staining in patients' (II-I, II-3) and controls fibroblasts to monitor mitochondrial membrane potential (MMP). Patients' cells display the release of free JC1 molecules (green), highlighting a mild dissipation of MMP.

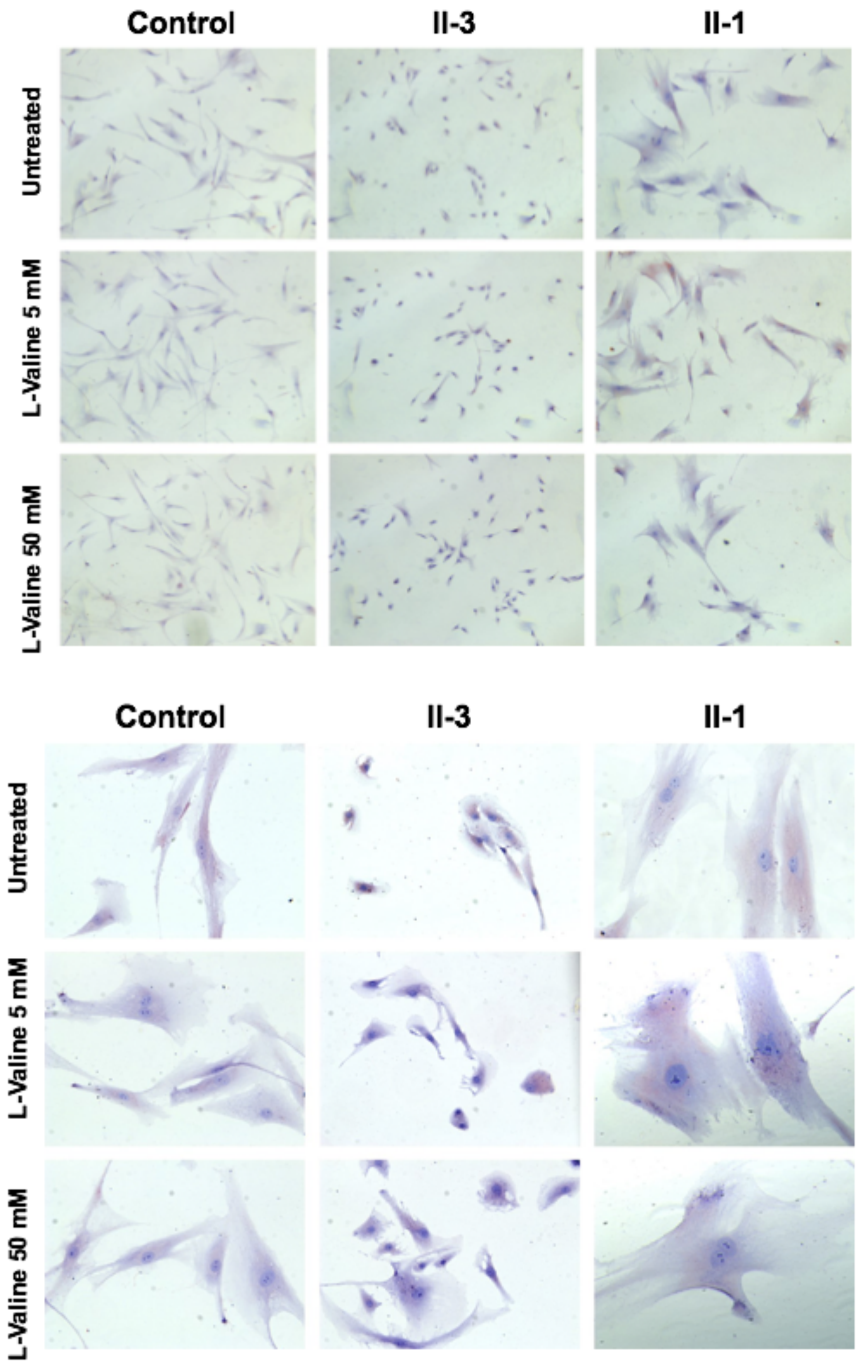

SUPPLEMENTARY FIGURE 9 Oil Red O Solution staining in control and patients' fibroblasts. Lipid accumulation (red dots) was not detected in cells cultured in standard condition or after the addition of increasing concentration of L-Valine (5 mM and 50 mM, 24 hours), regardless of their genotype.

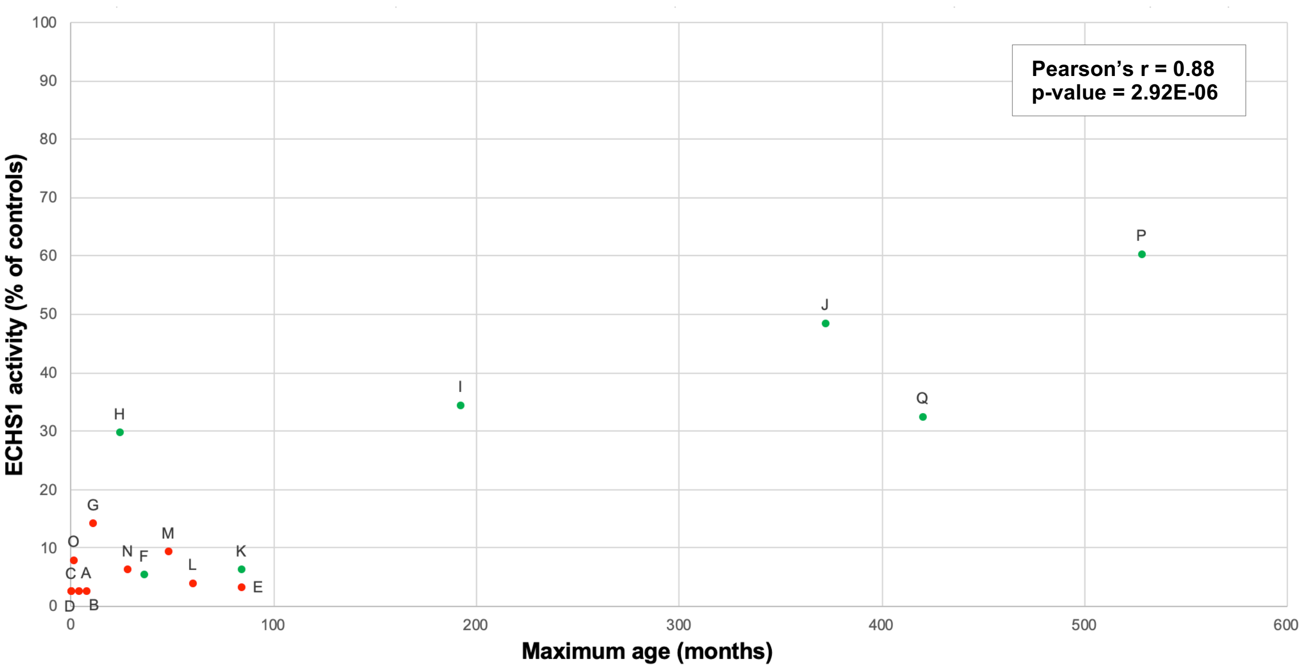

ID	Allele 1	Allele 2	Age Onset	Age Max	Status	% activity	Reference	
A	c.473C>A (p.Ala158Asp)	c.414+3G>C (r.spl)	Birth	4 m	dead	2.4	Sibling 1	Peters et al., 2014 [ref. 2]
B	c.473C>A (p.Ala158Asp)	c.414+3G>C (r.spl)	Birth	8 m	dead	2.4	Sibling 2	
C	c.817A>G (p.Lys273Glu)	c.817A>G (p.Lys273Glu)	Birth	24 h	dead	2.4	Patient 1	Ferdinandusse et al., 2014 [ref. 9]
D	c.817A>G (p.Lys273Glu)	c.817A>G (p.Lys273Glu)	Birth	2 d	dead	2.4	Patient 2	
E	c.433C>T (p.Leu145Phe)	c.476A>G (p.Gln159Arg)	4 m	7 y	dead	3.2	Patient 3	
F	c.673T>C (p.Cys225Arg)	c.674G>C (p.Cys225Ser)	Birth	3 y	alive	5.3	Patient 4	
G	c.197T>C (p.Ile66Thr)	c.449A>G (p.Asp150Gly)	Birth	11 m	dead	14.1	F2, II:1	Haack et al., 2015 [ref. 3]
H	c.673T>C (p.Cys225Arg)	c.673T>C (p.Cys225Arg)	Birth	2 y	alive	29.7	F5, II:3	
I	c.161G>A (p.Arg54His)	c.431dup (p.Leu145Alafs*6)	Birth	16 y	alive	34.4	F9, II:2	
J	c.229G>C (p.Glu77Gln)	c.476A>G (p.Glu159Arg)	11 m	31 y	alive	48.4	F10, II:1	
K	c.176A>G (p.Asn59Ser)	c.413C>T (p.Ala138Val)	10 m	7 y	alive	6.2	III-2	Yamada et al., 2015 [ref. 13]
L	c.176A>G (p.Asn59Ser)	c.413C>T (p.Ala138Val)	7 m	5 y	dead	3.9	III-3	
M	c.476A>G (p.Gln159Arg)	c.476A>G (p.Gln159Arg)	5 m	4 y	dead	9.2	Patient 1	Fitzsimons et al., 2018 [ref. 14]
N	c.538A>G (p.Thr180Ala)	c.538A>G (p.Thr180Ala)	5 m	28 m	dead	6.2	Patient 3	
O	c.8C>A (p.Ala3Asp)	c.836T>C (p.Phe279Ser)	Birth	40 d	dead	7.8	Patient	Bedoyan et al., 2017 [ref. 15]
P	c.244G>T (p.Val82Leu)	c.476A>G (p.Gln159Arg)	Birth	44 y	alive	60.3	II-1	This Study
Q	c.244G>T (p.Val82Leu)	c.476A>G (p.Gln159Arg)	Birth	35 y	alive	32.4	II-3	

SUPPLEMENTARY FIGURE 10 The chart shows *ECHS1*-mutated subjects for those the quantification of residual enzyme activity in fibroblasts (crotonyl-coA used as substrate) is available. Y-axis indicate the percentage of residual activity compared to controls used in the relative study. X-axis shows the maximum age reached, as reported. Red color indicates dead subjects. Green color indicates patients who were alive at the moment of their description. Pearson's correlation test indicates a positive (r = 0.88) and significant ($p<0.05$) correlation. Abbreviations: d (days), m (months), y (years).

SUPPLEMENTARY DATA REFERENCES

[S1] STERN JR, DEL CAMPILLO A, RAW I. Enzymes of fatty acid metabolism. I. General introduction; crystalline crotonase. J Biol Chem. 1956 Feb;218(2):971-83. PubMed PMID: 13295247.

[S2] Olgiati S, Skorvanek M, Quadri M, Minneboo M, Graafland J, Breedveld GJ, Bonte R, Ozgur Z, van den Hout MC, Schoonderwoerd K, Verheijen FW, van IJcken WF, Chien HF, Barbosa ER, Chang HC, Lai SC, Yeh TH, Lu CS, Wu-Chou YH, Kievit AJ, Han V, Gdovinova Z, Jech R, Hofstra RM, Ruijter GJ, Mandemakers W, Bonifati V. Paroxysmal exercise-induced dystonia within the phenotypic spectrum of ECHS1 deficiency. Mov Disord. 2016 Jul;31(7):1041-8. doi: 10.1002/mds.26610. Epub 2016 Apr 19. PubMed PMID: 27090768.

[S3] Kinkel AD, Fernyhough ME, Helterline DL, Vierck JL, Oberg KS, Vance TJ, Hausman GJ, Hill RA, Dodson MV. Oil red-O stains non-adipogenic cells: a precautionary note. Cytotechnology. 2004 Sep;46(1):49-56. doi:10.1007/s10616-004-3903-4. Epub 2005 Jun 16. PubMed PMID: 19003258; PubMed Central PMCID: PMC3449473.
